# Clinical features and prognosis of patients with anti-GBM disease combined with mesangial IgA deposition

**DOI:** 10.3389/fimmu.2024.1373581

**Published:** 2024-07-22

**Authors:** Wei Ning, Ya-fei Zhao, Ya-ru Liu, Yuan-yuan Qi, Zhan-zheng Zhao

**Affiliations:** ^1^ Department of Nephrology, The First Affiliated Hospital of Zhengzhou University, Zhengzhou, Henan, China; ^2^ Zhengzhou University, Zhengzhou, Henan, China; ^3^ Laboratory of Nephrology, The First Affiliated Hospital of Zhengzhou University, Zhengzhou, Henan, China

**Keywords:** anti-glomerular basement membrane disease, mesangial IgA deposition, renal prognosis, autoimmune disease, glomerulonephritis

## Abstract

**Introduction:**

Anti-GBM diseases with IgA deposition in the mesangial region are rarely described.The factors influencing renal prognosis in patients with anti-GBM disease combined with mesangial IgA deposition are unknown.

**Methods:**

We searched the pathological reports of the First Affiliated Hospital of Zhengzhou University from 2015 to 2023 and found that a total of 72 patients with the anti-GBM disease and 25 patients combined with mesangial IgA deposition. We studied the clinical and pathological features, renal prognosis, and the factors affecting renal prognosis in patients with anti-GBM disease combined with mesangial IgA deposition.

**Results:**

Their median age was 44 years, and their age distribution was unimodal. The proportion of oliguria or anuria in patients with anti-GBM disease combined with mesangial IgA deposition was significantly lower than that in patients with classic anti-GBM disease (13.04 vs. 42.31%, p=0.030). Their 24-hour urinary protein excretion was significantly higher [median:3.25 vs. 1.12g/24h, Interquartile range(IQR):1.032~3.945 vs. 0.63~1.79g/24h, p=0.020], serum creatinine (SCr) level at the initial diagnosis was lower(median:456.0 vs. 825.5μmol/L, IQR:270.0~702.0 vs. 515.8~1231.2μmol/L, p=0.002), peak SCr level was lower (median: 601.0 vs. 907.2μmol/L, IQR: 376.5~937.0 vs. 607.0~1361.2μmol/L, p=0.007), and their serum complement 3(C3) level was higher(median: 1.275 vs. 1.015g/L, IQR:1.097~1.462 vs. 0.850~1.220g/L, p=0.027). They had better renal outcomes during follow-up (p<0.001). After adjustment for hypertension, oliguria or anuria, and crescents%, IgA deposition in the mesangial region was still an independent protective factor (p=0.003) for ESRD in anti-GBM patients. Hypertension (p=0.026) and SCr levels at initial diagnosis (p=0.004) were risk factors for renal prognosis in patients with anti-GBM disease combined with mesangial IgA deposition.

**Discussion:**

Patients with anti-GBM disease combined with mesangial IgA deposition have less severe renal impairment and better renal prognosis than patients with classic anti-GBM disease.

## 1 Introduction

Anti-glomerular basement membrane disease(Anti-GBM disease) is an autoimmune disease in which the target antigen is present within a specific basement membrane, such as GBM and/or alveolar basement membrane. About 80 to 90 percent of patients will develop features of rapidly progressive glomerulonephritis. Pulmonary hemorrhage occurs in 40% to 60% of patients, and isolated lung disease may occur in a very small number of patients (1). Anti-GBM disease is rare, with an incidence of about 1.64 per million population per year (2). However, anti-GBM glomerulonephritis accounts for 10%–15% of all crescentic glomerulonephritis (3). This makes anti-GBM one of the most aggressive glomerular diseases.

Anti-GBM diseases with IgA deposition in the mesangial region are rarely described. Since 1998, when Trpkov, K. et al. first reported IgA deposition in the mesangial region with anti-GBM disease (4), 23 isolated case reports (4–26) and one case-control study (n=15) have been published (27). Some scholars believe that their kidney lesions are milder and their renal prognosis is significantly better than that of classical patients. However, due to the limited number of reported cases and the variable duration of follow-up, we still need to collect more cases to better understand the clinical, pathological, and prognostic data of anti-GBM disease combined with mesangial IgA deposition. The factors influencing renal prognosis in patients with anti-GBM disease combined with mesangial IgA deposition are unknown. So we searched the pathological reports of the First Affiliated Hospital of Zhengzhou University from 2015 to 2023 and found that a total of 72 patients with the anti-GBM disease and 25 patients combined with mesangial IgA deposition, accounted for 34.72%. We studied the clinical and pathological features, renal prognosis, and the factors affecting renal prognosis in patients with anti-GBM disease combined with mesangial IgA deposition.

## 2 Methods

### 2.1 Patients

**Figure 1:**
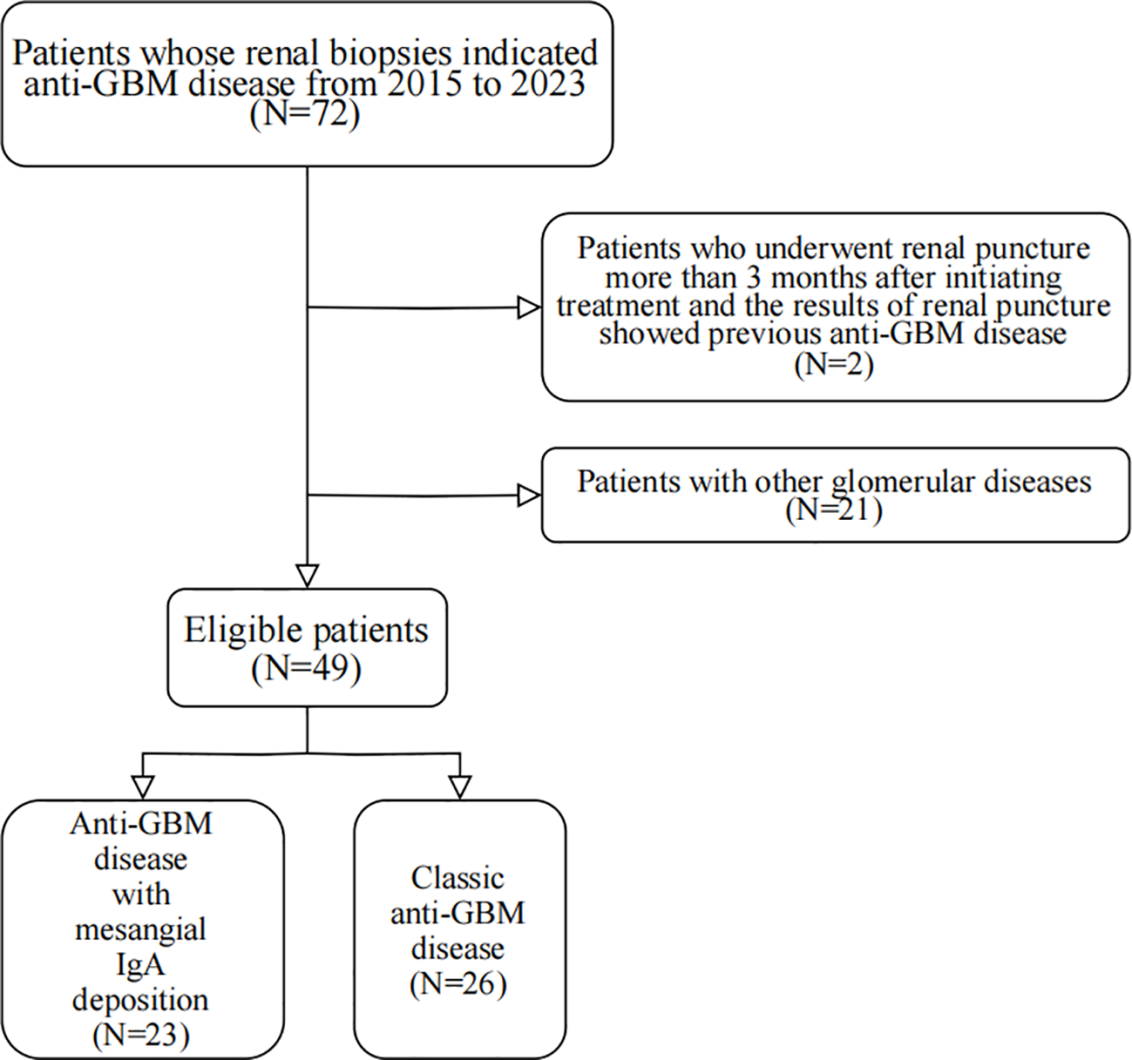
The flowchart of patient recruitment.

From 2015 to 2023, a total of 72 patients were retrieved according to the results of renal biopsy indicating anti-GBM disease, including 25 cases (34.72%) with mesangial IgA deposition and 47 cases (65.28%) without mesangial IgA deposition. Exclusion Criteria: 1. 2 patients who underwent renal puncture more than 3 months after initiating treatment and the results of renal puncture showed previous anti-GBM disease were excluded; 2. 21 patients with other glomerular diseases were excluded, including 19 cases with membranous nephropathy, 1 case with diabetic nephropathy, and 1 case with focal proliferative glomerulonephritis. The patient recruitment flowchart is shown in **Figure 1**. Approval was granted by the Ethics Committee of the First Affiliated Hospital of Zhengzhou University (Date2022-10-18/No2022-KY-1162-001).

### 2.2 Data acquisition

We collected demographic data, clinical symptoms, clinical test data, and renal pathological biopsy data at the time of patient presentation. We also collected data on kidney survival up to 82 months. To reduce bias, renal biopsy specimens were reviewed and analyzed separately by two renal pathologists and referred to a third specialist for comprehensive judgment in case of disagreement. End-stage renal disease (ESRD) is defined as dependence on renal substitution therapy for more than 3 months. Renal death is defined as the presence of ESRD.

### 2.3 Statistical analysis

Perform the Wilcoxon test or Kruskal-Wallis test for quantitative variables. Perform the Fisher test or the χ2 test for qualitative variables. Survival analysis was performed using the Kaplan-Meier method, univariate Cox regression method multivariate Cox regression method, and we used the LogRank test when necessary. Statistical analysis was performed using R software (version 4.3.1, https://cran.r-project.org). Some of the features are created by Microsoft Office (version 2311 Build 16.0.17029.20028).

## 3 Results

### 3.1 Patient features

For ease of explanation, we defined the group of patients with anti-GBM disease combined with mesangial IgA deposition as group A and the group of patients with classic anti-GBM disease as group B. The proportion of women in Group A was higher than in Group B, although it was not statistically significant (73.91 vs. 46.15%, p=0.075) (**Table 1**). The age distribution of Group A is unimodal with a peak around 45 years of age, and the age distribution of Group B is bimodal with the first peak occurring around 30 years of age and the second peak appearing around 65 years of age as shown in **Supplmentary Figure S1**.

**Table 1:** Demographic and clinical information of Group A and Group B.

Features	Group A (N=23) [% or median (IQR)]	Group B (N=26) [% or median (IQR)]	P-value
Demographic features			
Female	73.91	46.15	0.075
Age	44 (37.5~53.5)	48 (24~63.5)	0.652
Smoke	4.35	7.69	1
Clinical features			
Precursor infection	60.87	50.00	0.568
Gross Hematuria	34.78	46.15	0.588
Oliguria or anuria	13.04	42.31	0.030※
Hemoptysis	4.35	7.69	1
Pulmonary hemorrhage^a^	4.35	11.54	0.608
Hypertension	56.21	46.15	0.582
Diabetes	4.35	0.00	0.464
Dyslipidemia	17.39	7.69	0.390
ANCA^b^	8.70	26.92	0.145
AKI^c^	39.13	57.69	0.248
NS^d^	26.09	19.23	0.723
Treatment			
Initial dialysis treatment	69.57	92.31	0.064
Number of Plasmapheresis [times, median (IQR)]	6 (3~10)*	6 (2.75~9)*	0.537
Plasmapheresis	91.30	84.62	0.665
pulse methylprednisolone	100	84.62	0.119
Oral glucocorticoids	100	92.31	0.504
Cyclophosphamide	65.22	50.00	0.394
Rituximab	0.00	11.54	0.240
Mycophenolate mofetil	13.04	11.54	1
ICU^e^	13.04	34.62	0.108
Event			
ESRD	34.78	80.76	0.226
All-cause death	0	3.85	1
Clinical tests			
microscopic hematuria	100	95.83*	1
24-hour urinary protein excretion (g/24 h)^f^	3.25 (1.032~3.945)*	1.12 (0.63~1.79)	0.020※
Mean 24-hour urinary protein excretion from 0 to 3 months (g/24 h)^g^	1.788 (1.410~2.484)*	2.32 (2.150~4.005)*	0.441
Mean 24-hour urinary protein excretion from 3 to 6 months (g/24 h)	2.205 (1.477~3.307)*	1.155 (0.42~1.919)*	0.075
Mean 24-hour urinary protein excretion from 6 to 12 months (g/24 h)	2.12 (0.920~3.020)*	1.15 (0.825~1.340)*	0.172
Mean 24-hour urinary protein excretion from 12 months to the end of follow-up (g/24 h)	2.03 (0.609~2.700)*	0.405 (0.298~0.565)*	0.092
SCr (μmol/L)^h^	456 (270~702)	825.5 (515.8~1231.2)	0.002**※**
Peak SCr (μmol/L)^i^	601 (376.5~937)	907.2 (607~1361.2)	0.007**※**
eGFR (ml/min/1.73m^2^)^j^	10.856 (5.75~22.006)	4.92 (3.528~8.214)*	0.004**※**
WBC (×10^9/L)^k^	9.00 (7.657~10.812)*	9.38 (7.857~11.80)*	0.425
Hb (g/L)^l^	90 (80~95.6)	83 (71.25~95)*	0.356
Alb (g/L)^m^	32.5 (28.75~34.5)	30.45 (26.85~34.35)*	0.597
Serum C3 (g/L)^n^	1.275 (1.097~1.462)*	1.015 (0.85~1.22)*	0.027**※**
Serum C4 (g/L)^o^	0.305 (0.2775~0.3675)*	0.26 (0.23~0.30)*	0.223
ESR (mm/h)^p^	114 (70.00~127.00)*	80 (29.50~123.00)*	0.296
CRP (mg/L)^q^	54.7 (14.38~87.53)*	38.67 (9.06~116.92)*	1
Serum IgA (g/L)^r^	3.12 (2.223~3.410)*	1.92 (1.415~2.962)*	0.070
Serum IgG (g/L)^s^	11.30 (9.485~16.15)*	10.38 (8.09~13.01)*	0.202
Serum IgM (g/L)^t^	1.195 (0.7425~1.4875)*	1.015 (0.725~1.238)*	0.499
Anti-GBM (U/mL)^u^	560 (247.5~712.5)	509 (392.5~694.5)	0.804
Time for anti-GBM antibodies to change from positive to negative (month)	1.467 (0.850~2.817)*	1.200 (0.667~2.400)*	0.878
Positive rate of anti-GBM antibodies at 1 month after diagnosis	70.000	64.706	1
Positive rate of anti-GBM antibodies at 2 months after diagnosis	31.579	46.667	0.476
Positive rate of anti-GBM antibodies at 3 months after diagnosis	26.316	15.385	0.670
Positive rate of anti-GBM antibodies at 6 months after diagnosis	0.000	0.000	0.377

※P<0.05;

*The project is missing information about some of its patients;

a Pulmonary hemorrhage: the presence of hemoptysis or the presence of bleeding in bronchoscopic lavage or chest CT showing interstitial opacity;

b ANCA:Patients combined with ANCA-associated vasculitis;

c AKI: patients combined with acute kidney injury;

d NS: patients combined with nephrotic syndrome;

e ICU: patients admitted to the intensive care unit during follow-up;

f 24-hour urinary protein excretion (g/24 h): 24-hour urinary protein excretion at initial diagnosis;

g Mean 24-hour urinary protein excretion from 0 to 3 months (g/24 h): 24-hour urinary protein excretion at initial diagnosis was not included;

h SCr: Serum creatinine level at initial diagnosis;

i Peak SCr: Peak serum creatinine of the patient during follow-up;

j eGFR: estimated glomerular filtration rate;

k WBC: White blood cell count;

l Hb: hemoglobin;

m Alb: serum albumin;

n Serum C3: Serum complement 3; Normal values < 1.57g/L;

o Serum C4: Serum complement 4; Normal values < 0.44g/L;

p ESR: Erythrocyte Sedimentation Rate; Normal values <15mm/h;

q CRP: C-reaction protein; Normal values <10mg/L;

r Serum IgA: Normal values <4.53mg/L;

s Serum IgG: Normal values <14.25mg/L;

t Serum IgM: Normal values <3.04g/L;

u Anti-GBM: Anti-GBM antibody quantification; Normal values <100U/mL;

Symptoms of Group A onset include precursor infection (60.87%), oliguria or anuria (13.04%), gross hematuria (34.78%), and hemoptysis (4.35%). 13 people in Group A had hypertension (56.52%) (**Table 1**). 2 people in Group A had ANCA-associated vasculitis (8.70%). 9 people in Group A developed acute kidney injury (AKI, 39.13%). In Group A, 21 received plasmapheresis (91.30%), 23 received pulse methylprednisolone (100%), 15 received cyclophosphamide (65.22%), 3 received mycophenolate mofetil (13.04%), and 0 received rituximab. During follow-up (Median follow-up time: 21.6 months), 9 in Group A progressed to ESRD (34.78%) and 0 died (**Table 1**).

The proportion of oliguria or anuria in Group A was significantly lower than that in Group B, and the difference was statistically significant(13.04 vs. 42.31%, p=0.030) (**Table 1**). In addition, the proportion of people who received renal replacement therapy on initial admission of Group A was lower than that of Group B, although it was not statistically significant(69.57 vs. 92.31%, p=0.064) (**Table 1**).

The 24-hour urinary protein excretion of Group A was significantly higher than that of Group B at the time of initial admission [median:3.25 vs. 1.12g/24h, Interquartile range(IQR):1.032~3.945 vs. 0.63~1.79g/24h, p=0.020] (**Table 1**). We followed up on patients’ 24-hour urinary protein excretion in Groups A and B. We calculated the mean of 24-hour urinary protein excretion of each patient from 0 to 3 months, from 3 to 6 months, from 6 to 12 months, and from 12 months to the end of follow-up. We found no significant difference in mean 24-hour urinary protein excretion from 0 to 3 months between Group A and Group B (median: 1.788vs2.320g/24h, IQR: 1.410~2.484 vs 2.150~4.005g/24h, p=0.441) (**Table 1**). Although the medians of the means of 24-hour median urinary protein excretion from 3 to 6 months, from 6 to 12 months, and from 12 months to the end of follow-up in group A were greater than those in group B, there were no statistical differences between Group A and Group B(from 3 to 6 months: median: 2.205 vs 1.155g/24h, IQR: 1.477~3.3.7 vs 0.420~1.919g/24h, p=0.075; from 6 to 12 months: median: 2.120 vs 1.150g/24h, IQR: 0.920~3.020 vs 0.825~1.340g/24h, p=0.172; from 12 months to the end of follow up: median:2.030 vs 0.405, IQR: 0.609~2.700 vs 0.298~0.565g/24h, p=0.092)(**Table 1**).

The serum creatinine (SCr) level of Group A was significantly lower than that of Group B at the initial diagnosis (median:456.0 vs. 825.5μmol/L, IQR:270.0~702.0 vs. 515.8~1231.2μmol/L, p=0.002) (**Table 1**). The peak SCr level of group A was significantly lower than that of group B (median: 601.0 vs. 907.2μmol/L, IQR:376.5~937.0 vs. 607.0~1361.2μmol/L, p=0.007) (**Table 1**). The estimated glomerular filtration rate (eGFR) of Group A was statistically significantly higher than that of Group B (median:10.856 vs. 4.920ml/min/1.73m^2^, IQR:5.750~22.006 vs. 3.528~8.210ml/min/1.73m^2^, p=0.004) (**Table 1**). The serum complement3 (C3) level of Group A was statistically significantly higher than that of Group B (median: 1.275 vs. 1.015g/L, IQR:1.097~1.462 vs. 0.850~1.220g/L, p=0.027) (**Table 1**). Although not statistically significant, the serum immunoglobulin A(IgA) level of group A was higher than that of group B (median: 3.12 vs. 1.92g/L, IQR:2.223~3.410 vs. 1.415~2.962g/L, p=0.070) (**Table 1**).

There was no statistically significant difference between Group A and Group B regarding pulmonary hemorrhage and comorbid ANCA-associated vasculitis (**Table 1**). There was no statistically significant difference in the levels of anti-GBM antibody between Group A and Group B at initial diagnosis(median: 560 vs 509U/mL, IQR: 247.5~712.5 vs 392.5~694.5U/mL, p=0.804), and no statistically significant difference in the time of anti-GBM antibody positive to negative between Group A and Group B(median: 1.467 vs 1.200months, IQR: 0.850~2.817 vs 0.667~2.400months, p=0.878) (**Table 1**). Some patients were not observed to change from positive to negative in anti-GBM antibodies before the loss of follow-up and were not included in statistical analysis. We followed up the anti-GBM antibody levels of the patients at 1, 2, 3, and 6 months after diagnosis, and found no statistically significant difference in the composition of antibody positive between group A and group B (1 month: 70.000% vs 64.706%, p=1;2 months: 31.579% vs 46.667%, p=0.476; 3 months: 26.316% vs 15.385%, p=0.67; 6months: 0% vs 0%, p=0.377) (**Table 1**).

**Figure 2:**
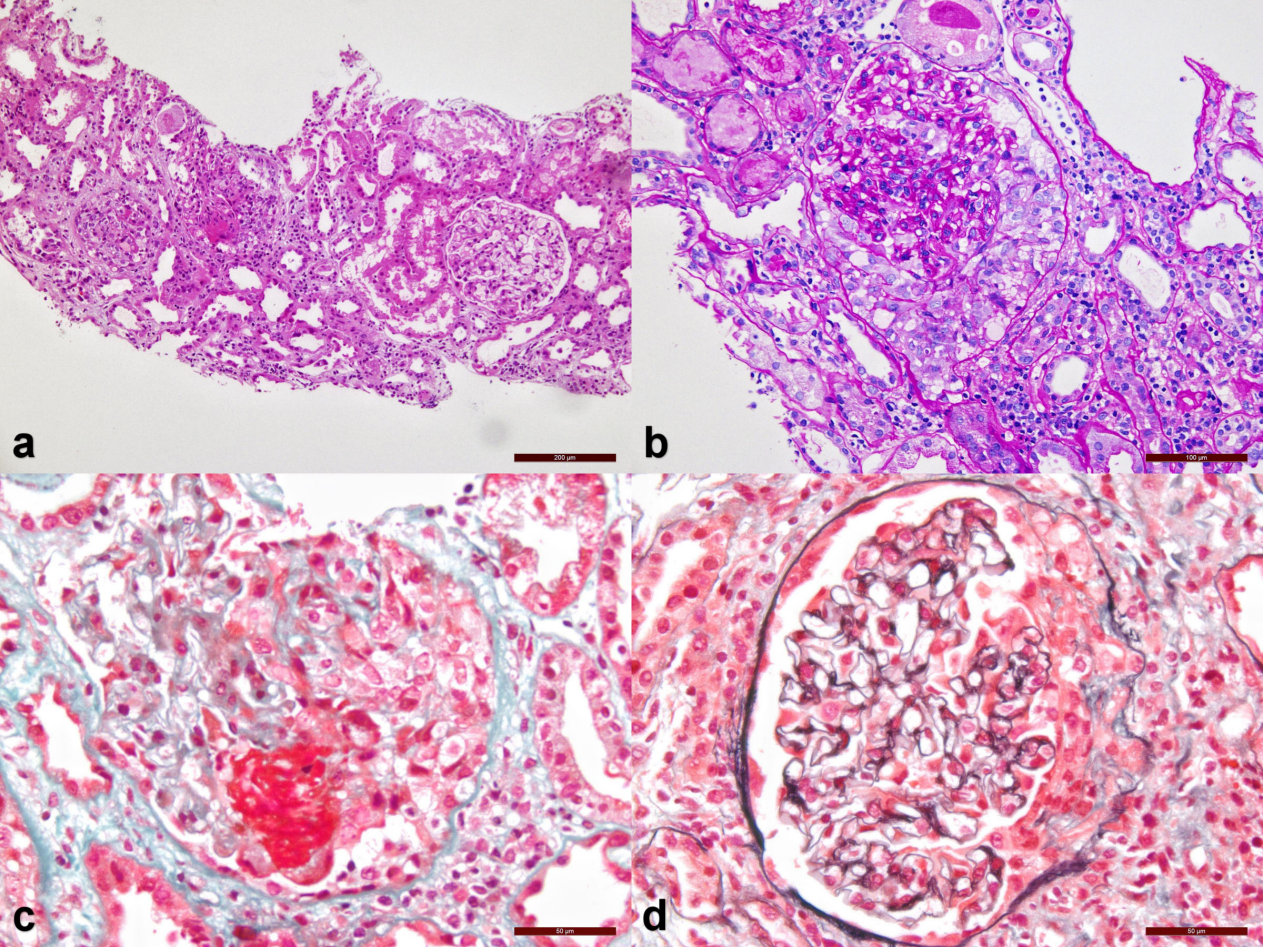
Pathological images of Group A **(A)** Hematoxylin-eosin staining, magnification 100 times; **(B)** Periodic Acid-Schiff staining, magnification 200 times; **(C)** Masson staining, magnification 400 times; **(D)** P+M staining, magnification 400 times.

In the renal biopsy results, Group A was statistically different from Group B in electron-dense deposit (73.68 vs. 0%, p<0.001). Although not statistically significant, the proportion of renal interstitial fibrosis was higher in Group A than in Group B(p=0.090) (**Table 2**). We also collected some representative pathological images of group A patients (**Figure 2**). Under the optical microscope, we can see 1 renal cortex, 2 renal cortex medullary junctions, 1 renal medulla, and 20 glomeruli. We can see the mild proliferation of mesangial cells and stroma. We can see 6 loop necrosis (4 with cellular Crescents), 4 with cellular crescents, 2 with cellular fibrous crescents (1 with rupture of Bowman’s capsule wall), and 2 with small cellular crescents. We can see vacuolar degeneration and granular degeneration of renal tubular epithelial cells. We can see sheet-like monocytes, lymphocytes, and plasma cells infiltrating the renal interstitium (**Figure 2**).

**Table 2:** Pathological features of Group A and Group B.

Features	Group A (N=23)[median (IQR)]	Group B (N=26)[median (IQR)]	P-value
IgG deposit intensity (scale 0~3+)	2.0 (0.5~2.0)	2.0 (0.0~3.0)	0.199
IgG1 deposit intensity (scale 0~3+)	1.5 (0.0~2.5)*	2.0 (0.0~3.0)*	0.382
IgG2 deposit intensity (scale 0~3+)	1.0 (0.0~2.0)*	1.0 (0.0~2.0)*	0.842
IgG3 deposit intensity (scale 0~3+)	0.25 (0.0~1.00)*	0.00 (0.00~2.0)*	0.726
IgG4 deposit intensity (scale 0~3+)	0.5 (0.0~2.0)*	0.0 (0.0~2.0)*	0.237
IgA deposit in mesangium (scale 0~3+)	2.0 (1.5~3.0)	0.0	<0.001**※**
IgM deposit intensity (scale 0~3+)	1.0 (0.0~2.0)*	1.0 (0.0~2.5)*	0.708
C3 deposit intensity (scale 0~3+)	2.0 (0.0~3.0)	1.5 (0.0~2.0)	0.471
C4 deposit intensity (scale 0~3+)	0.0 (0.0~1.0)*	0.0 (0.0~1.0)*	0.571
C1q deposit intensity (scale 0~3+)	0.0 (0.0~1.0)*	0.0 (0.0~2.0)*	0.398
FRA deposit intensity (scale 0~3+)	0.0 (0.0~1.0)*	1.0 (0.0~2.0)*	0.203
κ deposit intensity (scale 0~3+)	1.0 (0.0~2.0)	1.0 (0.0~2.0)*	0.676
λ deposit intensity (scale 0~3+)	1.0 (0.0~2.5)	1.5 (0.0~2.0)*	0.714
Number of glomeruli	20.0 (7~67)	18.5 (4~47)	\
Glomerular sclerosis	0.00 (0~32.84)	1.19 (0~38.10)	0.532
Cellular crescents	50.00 (0~90.48)	45.05 (0~97.62)	0.810
Fibrocellular crescents	17.65 (0~50.00)	12.20 (0~78.57)	0.718
Fibrous crescents	7.14 (0~46.27)	4.38 (0~100.00)	0.836
Crescents	87.50 (30.0~100.0)	92.72 (58.7~100.0)	0.162
Tubular atrophy	86.96	80.77	0.710
Renal interstitial fibrosis	47.83	73.08	0.090
Electron-dense deposit	73.68*	0.00*	<0.001**※**

*The project is missing information about some of its patients;

※P<0.05;

“\” indicates that statistical analysis is not necessary.

### 3.2 Kidney survival

**Figure 3:**
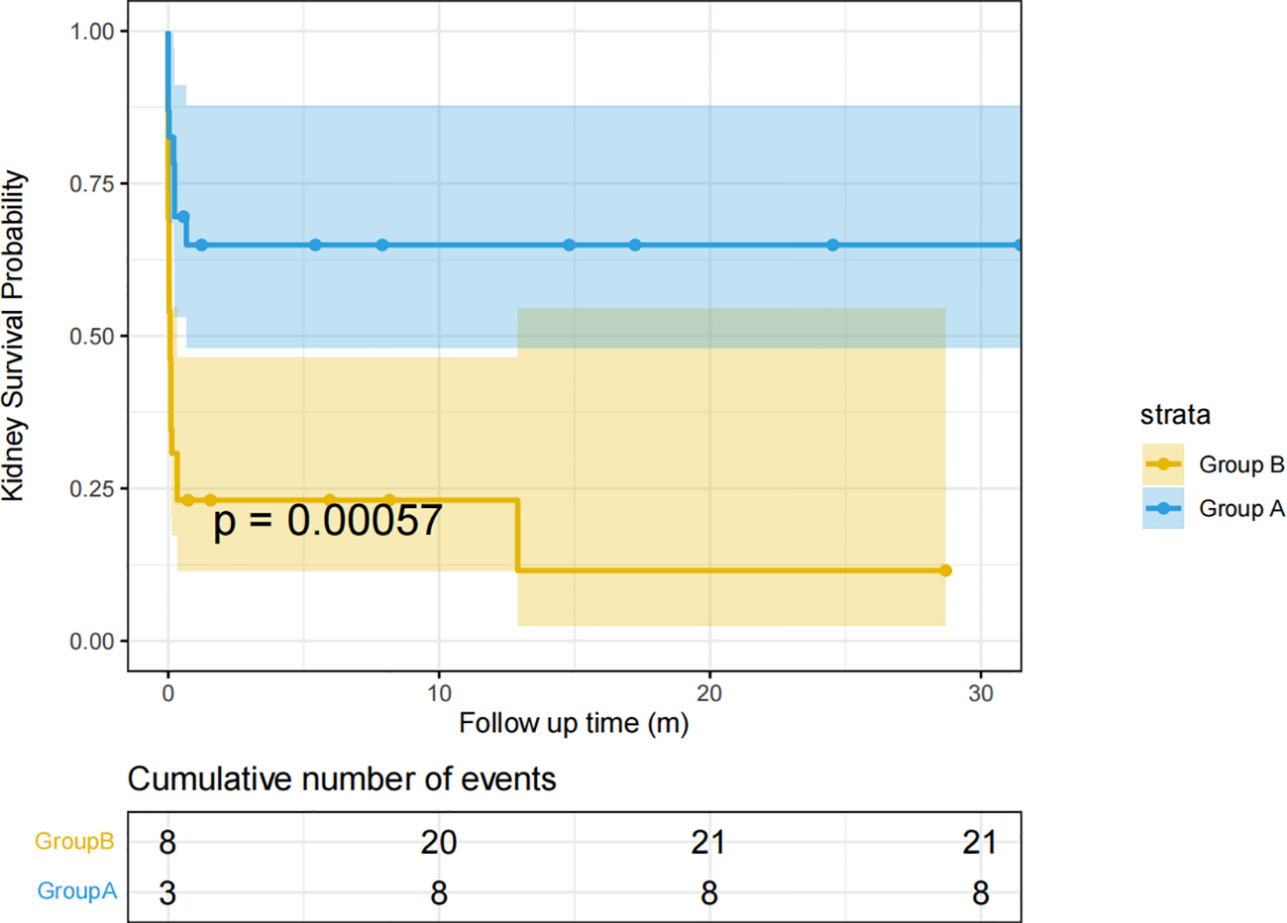
Renal survival curves of groups A and B Kidney survival time (months) is defined as the time from the diagnosis of the disease to the onset of ESRD or the last follow-up.

The survival curves of Group A and Group B are shown in **Figure 3**. 21 (80.76%) in Group B had renal death at follow-up. However, only 8 (34.78%) in Group A had renal death. The renal prognosis of Group A was significantly better than Group B(p<0.001) (**Figure 3**).

### 3.3 Predictors of ESRD in anti-GBM disease

Univariate analysis showed that the intensity of IgA deposition in the mesangial region was significantly associated with the risk of ESRD (HR: 0.51, 95%CI: 0.34~0.77, p=0.001) (**Table 3**). In addition, the univariate analysis also showed that smoking, hypertension, oliguria or anuria, SCr, hemoglobin (Hb), serum IgA, deposit intensity of IgG1 and IgG3 in kidney biopsies, fibro cellular crescents%, crescents%, and electron-dense deposit were significantly associated with the risk of ESRD (p<0.05) (**Table 3**). After adjustment for hypertension, oliguria or anuria, and crescents%, multivariate analysis still showed that the intensity of IgA deposit in the mesangial was an independent protective factor for ESRD(**Table 3**). For every 1+ increase in the intensity of IgA deposit in the mesangial region, the risk of ESRD decreases by 48% (HR: 0.52, 95%CI: 0.34~0.80, p=0.003) (**Table 3**). In addition, hypertension and oliguria or anuria were also associated with ESRD in multivariate analysis (p<0.05) (**Table 3**).

**Table 3:** Predictors of ESRD by univariate and multivariate Cox regression analysis.

Variables	Univariate analysis (N = 49)		Multivariate analysis^v^ (N = 49)	
	HR (95% CI)	p-value	HR (95% CI)	p-value
Smoking (0=no,1=yes)	2.99 (0.88~10.13)	0.079	\	\
Hypertension (0=no,1=yes)	2.47 (1.14~5.32)	0.021**※**	3.15 (1.39~7.16)	0.006**※**
Oliguria or anuria (0=no,1=yes)	3.11 (1.47~6.60)	0.003**※**	3.80 (1.67~8.68)	0.002**※**
SCr (increased by 100μmol/L)	1.1 (1.05~1.16)	<0.001※	\	\
Hb (n=47,increased by 10g/L)	0.76 (0.61~0.96)*	0.018※	\	\
Serum IgA (n=32, increased by 1.0g/L)	0.57 (0.37~0.87)*	0.010※	\	\
IgG1 deposit intensity (n=47,increased by 1+)	1.62 (0.94~2.81)*	0.084	\	\
IgG3 deposit intensity (n=47,increased by 1+)	2.47 (1.17~5.23)*	0.018※	\	\
IgA deposit intensity (increased by 1+)	0.51 (0.34~0.77)	0.001**※**	0.52 (0.34~0.80)	0.003**※**
Crescents (increased by 10%)	1.44 (1.07~1.94)	0.016**※**	1.29 (0.94~1.79)	0.118
Fibrocellular crescents (increased by 10%)	1.2 (1.01~1.42)	0.036※	\	\
electron-dense deposit (n=38,0=no,1=yes)	0.10 (0.02~0.44)*	0.002※	\	\

v The hazard ratio (HR) of multivariate analysis was adjusted for hypertension, oliguria or anuria, IgA deposit intensity, and crescents%. The multivariate Cox regression model shown in the table is the optimal model after screening for independent variables.

※P<0.05;

*The project is missing information about some of its patients;

“\” indicates that these variables were not included in the multivariate cox regression model after independent variable screening.

### 3.4 Predictors of ESRD in anti-GBM disease combined with mesangial IgA deposition

Univariate COX analysis showed that smoking (HR:17.05, p=0.023), SCr (HR:1.29, p<0.001), and Hb (HR:0.55, p=0.021) were associated with renal prognosis in patients of Group A (**Table 4**). Variable selection was performed on the variables in **Table 4** to obtain the best multivariate COX regression model. In this model, hypertension (p=0.026) and SCr (p=0.004) were risk factors for renal death in patients of Group A (**Table 4**).

**Table 4:** Predictors of ESRD of Group A.

Variables	Univariate analysis (N = 23)		Multivariate analysis^w^ (N = 23)	
	HR (95% CI)	p-value	HR (95% CI)	p-value
Smoking (0=no,1=yes)	17.05 (1.48~196.90)	0.023**※**	\	\
Hypertension (0=no,1=yes)	7.52 (0.92~61.41)	0.060	55.05 (1.63~1859.14)	0.026**※**
SCr (increased by 100umol/L)	1.29 (1.11~1.48)	<0.001**※**	7.21 (1.87~27.86)	0.004**※**
Hb (increased by 10g/L)	0.55 (0.33~0.91)	0.021※	\	\

w The multivariate Cox regression model shown in the table is the optimal model after screening for independent variables.

※P<0.05;

“\” indicates that these variables were not included in the multivariate cox regression model after independent variable screening.

## 4 Discussion

Our results suggest that patients with anti-GBM disease combined with mesangial IgA deposition have higher 24-hour urine protein excretion at diagnosis (p=0.02), less incidence of oliguria or anuria (p=0.03), lower SCr levels at diagnosis(p=0.002), and better renal prognosis (p<0.001) than patients with classical anti-GBM disease. After adjustment for hypertension, oliguria or anuria, and crescents%, IgA deposit in the mesangial was still an independent protective factor (p=0.003) for ESRD in anti-GBM patients. Hypertension (0.026), and SCr level at diagnosis (0.004) were risk factors for renal prognosis in patients with anti-GBM disease combined with mesangial IgA deposition.

It seems paradoxical that patients with anti-GBM disease combined with IgA deposition in the mesangial region have higher urinary protein excretion at diagnosis but better renal outcomes. Our results showed that patients with anti-GBM disease combined with IgA deposition in the mesangial region have no statistical difference in the treatment of classical anti-GBM patients (Initial dialysis treatment: p=0.06; Number of plasma exchanges:p=0.537; Plasmapheresis: p = 0.665; pulse methylprednisolone:p=0.119; Oral steroid:p=0.504; Cyclophosphamide: p = 0.394; Rituximab, p = 0.24; Mycophenolate mofetil:p=1). We think that this may be related to better response to treatment in patients combined with mesangial IgA deposition, which is also suggested by the case report of F. Shaojie et al. (22), and the clinical study of C. R. Shen et al. (27). We followed up the 24-hour urinary protein excretion between the two groups, and the results showed no statistical difference in the mean 24-hour urinary protein excretion between the two groups from 0 to 3 months, 3 to 6 months, 6 to 12 months, and 12 months to the end of follow-up. We found that the median and quartile of the mean 24-hour urinary protein excretion from 0 to 3 months after diagnosis decreased compared with the 24-hour urinary protein excretion at diagnosis in patients with anti-GBM disease combined with IgA deposition in the mesangial region. The median and quartile of the mean 24-hour urinary protein excretion from 0 to 3 months after diagnosis increased compared with the 24-hour urinary protein excretion at diagnosis in patients with classical anti-GBM disease. However, due to the absence of data and the failure of the normality test, we could not conduct a T-test or ANOVA of repeated measurement design to obtain statistically significant conclusions. According to the follow-up results, we think patients with anti-GBM disease combined with IgA deposition in the mesangial region may achieve good alleviating proteinuria at the early stage of treatment, while patients with classical anti-GBM disease still have proteinuria progression at the early stage of treatment. The kidney survival curves of the two groups also supported this conclusion. We can observe that there is a large gap in renal survival between the two groups at the early stage of follow-up, and the renal survival rate of patients with anti-GBM disease combined with mesangial IgA deposition is significantly higher than that of classical anti-GBM disease. We speculate that this may be one of the characteristics of the anti-GBM disease combined with IgA deposition in the mesangial region. As for the mechanism of high urinary protein excretion at diagnosis, we hypothesize that it may be related to renal injury due to pathogenic immune complex deposition in the mesangium (28). One of the known pathogenesiss of IgA nephropathy is the aggregation of circulating immune complexes in the mesangium of the glomerulus, which induces cellular inflammation and damage, leading to increased urinary protein excretion (29). It may be that basal membrane inflammation caused by anti-GBM disease combined with mesangial inflammation caused by IgA nephropathy leads to higher urinary protein excretion. The inflammation could be reversed after treatment initiation, which may explain the decreased urinary protein excretion in patients with anti-GBM disease combined with mesangial IgA deposition at the early stage of treatment.

Patients with anti-GBM disease combined with mesangial IgA deposition have less incidence of oliguria or anuria, lower SCr levels at diagnosis, and better renal outcomes than patients with the classical anti-GBM disease, which indicates less impairment of kidney function. Zhao J et al. thought that IgG1 and IgG3 subclasses may play a crucial role in the pathogenesis of anti-GBM diseases (30). C. R. Shen et al. suggested that the lower prevalence of circulating anti-α3 (IV)NC1 IgG1 and IgG3 subclasses in patients with anti-GBM disease combined with IgA nephropathy(IgAN) may be associated with milder renal disease (27). We studied the subclasses of IgG antibodies deposited along the glomerular basement membrane between patients with anti-GBM disease combined with mesangial IgA deposition and patients with the classical anti-GBM disease and found that there was no statistical difference in the deposition of each subclass (IgG1: p=0.382; IgG2: p=0.842; IgG3: p=0.726; IgG4:p=0.237). Whether the presence and action of IgG1 and IgG3 anti-GBM antibodies are related to better renal prognosis in patients with mesangial IgA deposition needs further experimental verification.

It is not clear whether mesangial IgA deposition or anti-GBM disease occurs first, or whether it occurs simultaneously so far. We hypothesize that IgA deposition in the mesangial region precedes anti-GBM disease. Current research results suggest that pathogenic circulating IgA1-IgG immune complexes in patients with IgA nephropathy enter the renal circulation and are deposited in the mesangium of the glomeruli, resulting in mesangial cell proliferation and expansion of extracellular matrix components. This immune complex has a high affinity for fibronectin, the extracellular matrix components in the mesangium, and type IV collagen (28). The main target antigen of anti-GBM autoantibodies is the non-collagen domain (NC1) of the α3 chain of type IV collagen [α3(IV)NC1], and the content of a3 (IV) in the basement membrane is tissue-specific, with the highest content of a3 (IV) in the basement membrane of patients with anti-GBM (31). We hypothesize that the pathogenesis of patients with anti-GBM disease and IgA deposition in the mesangial region may be as follows: the patient begins with IgA deposition in the mesangial region, and the pathogenic circulating immune complex binds to type IV collagen in the mesangium of the glomeruli, changing the conformation of type IV collagen, exposing the α3 chain, and the patient produces anti-a3 (IV) antibodies, i.e., anti-GBM antibodies, which leads to the development of anti-GBM disease. Matsuno et al. observed that the titer of anti-GBM antibodies changed from negative to positive in the patient with IgA nephropathy during the disease, suggesting the possibility of our view (26). In addition, two patients in the cohort of Shen, C. R., et al. had IgA nephropathy before the onset of anti-GBM disease (27). However, whether the anti-GBM disease in these patients is an incidental complication or secondary to IgA deposition in the mesangial region remains difficult to prove. More researches are needed.

We also summarized all cases of anti-GBM disease combined with mesangial IgA deposition published in Pubmed (http://www.ncbi.nlm.nih.gov/pubmed) to date (including our cohort, a total of 61 cases, since 1998), as shown in **Table 5** (4–26).

**Table 5:** Clinical characteristics of 61 cases (4–26) (including the present cohort) of anti-GBM with mesangial IgA deposition in the literature.

Characteristics	% or Median (IQR)
Demographic features	
Female	63.93
Age	44.50 (34.00~54.25)
Smoke	18.18*
Clinical features	
Precursor infection	46.43*
Gross Hematuria	47.54
Oliguria or anuria	20.00
Hemoptysis or abnormal chest findings	3.28*
ANCA	4.91
24-hour urinary protein excretion (g/24h)	2.45 (1.17~4.00)*
SCr (g/L)	400.45 (255.00~699.90)
Serum C3(g/L)^x^	1.21 (0.85~1.44)*
Hb(g/L)	93.00 (83.00~99.00)*
Anti-GBM(U/mL)^y^	200.00 (126.00~552.00)*
Pathological features	
IgA deposit	100
Glomerular sclerosis	3.00 (0.00~17.71)*
Crescents	77.07 (60.5~90.68)*
Tubular atrophy	86.67*
Renal interstitial fibrosis	65.22*
Electron-dense deposit	81.63*
Treatment	
Initial dialysis treatment	52.73
Plasmapheresis	85.00*
Methylprednisone pulse therapy	98.33*
Immunosuppressants^z^	84.75*
Rituximab	6.82*
Event	
ESRD	39.29
Die	1.64

*The project is missing information about some of its patients;

x Serum C3< 1.57 g/L;

y Anti-GBM, Anti-GBM antibody quantification; Due to the different kits used to detect anti-GBM antibodies, we only analyzed the antibody titers measured by kits with normal values of less than 100 U/ml.

z Immunosuppressants include Cyclophosphamide, Mycophenolate mofetil, and Methotrexate.

The median age of all reported patients with anti-GBM disease combined with mesangial IgA deposition was 44.5 years, and 63.93% of patients were female (**Table 5**). The age distribution of patients showed an unimodal distribution, as shown in **Supplementary Figure S2**. The unimodal age distribution may suggest the order in which the disease occurs. The male-to-female ratio was approximately 1:1.77, suggesting that estrogen levels may be related to the occurrence of the anti-GBM disease combined with mesangial IgA deposition. Of the 61 patients, 48 were in China, 4 in Japan, 3 in India, 2 in the United States, 2 in Australia (1 in Asian, 1 Caucasian), 1 in South Korea, and 1 in Canada. This suggests that the incidence of anti-GBM disease combined with mesangial IgA deposition may vary greatly from region to region.

18% of patients smoked (**Table 5**). Precursor infection precedes onset in 43% of patients. Gross hematuria occurs in 45% of patients(**Table 5**). Oliguria or anuria occurs in 45% of patients (**Table 5**). 2 patients (3.28%) developed pulmonary hemorrhage (**Table 5**). 3 patients (4.91%) had ANCA-associated vasculitis (**Table 5**). The 24-hour urine protein excretion, SCr level, median serum C3, and hemoglobin values were 2.45 g/24 h, 400.45 μmol/L, 1.21 g/L, and 93 g/L, respectively (**Table 5**). The median anti-GBM antibody titer was 200 U/mL (**Table 5**). In all cases, pathological findings showed mesangial IgA deposition. The median rates of glomerular sclerosis and crescent formation were 3% and 77.07%, respectively. In addition, 86.67% of the patients had tubular atrophy, 65.22% had renal interstitial fibrosis, and 81.63% had electron-dense deposition (**Table 5**). Of all patients, 52.73% received initial dialysis therapy, 85% received plasmapheresis, 98.33% received pulse methylprednisolone, 84.75% received immunosuppressants, and 6.82% received rituximab (**Table 5**). In the end, 22 (39.39%) of patients progressed to ESRD, and 1 (1.64%) patient died (died of sepsis) (**Table 5**). This suggests that anti-GBM disease combined with mesangial IgA deposition may have a better renal prognosis.

There are certain limitations to our study. As our study is retrospective, we were unable to obtain sera samples from patients for investigation of IgG subclasses. Because the disease we studied is so rare, our sample size is small and we may not be able to draw very accurate conclusions. We hope that there will be more prospective studies with larger sample sizes in the future.

In summary, we report the largest cohort of the currently known anti-GBM disease combined with mesangial IgA deposition (n=23). We found that patients with anti-GBM disease combined with mesangial IgA deposition may have less kidney damage, better renal outcomes, and better response to treatment than patients with the classical anti-GBM disease. Anti-GBM patients combined with IgA deposition in the mesangial region need early detection and treatment to expect a better renal prognosis.

## 5 Conclusions

Patients with anti-GBM disease combined with mesangial IgA deposition have less kidney damage and better renal prognosis than patients with classic anti-GBM disease.

## Data availability statement

The original contributions presented in the study are included in the article/**Supplementary Material**. Further inquiries can be directed to the corresponding authors.

## Ethics statement

The studies involving humans were approved by the Ethics Committee of the First Affiliated Hospital of Zhengzhou University. The studies were conducted in accordance with the local legislation and institutional requirements. The participants provided their written informed consent to participate in this study. Written informed consent was obtained from the individual(s), and minor(s)’ legal guardian/next of kin, for the publication of any potentially identifiable images or data included in this article.

## Author contributions

WN: Data curation, Formal analysis, Investigation, Methodology, Validation, Visualization, Writing – original draft, Writing – review & editing. Y-FZ: Data curation, Investigation, Writing – review & editing. Y-RL: Data curation, Investigation, Writing – review & editing. Y-YQ: Conceptualization, Funding acquisition, Project administration, Resources, Supervision, Validation, Writing – original draft, Writing – review & editing. Z-ZZ: Conceptualization, Funding acquisition, Project administration, Resources, Supervision, Validation, Writing – original draft, Writing – review & editing.
