# Supplementary figures and images for: Clinical features and prognosis of patients with anti-GBM disease combined with mesangial IgA deposition
Source: Front Immunol. 2024 Jul 22;15:1373581. doi: 10.3389/fimmu.2024.1373581 (PMC11298365; doi:10.3389/fimmu.2024.1373581)

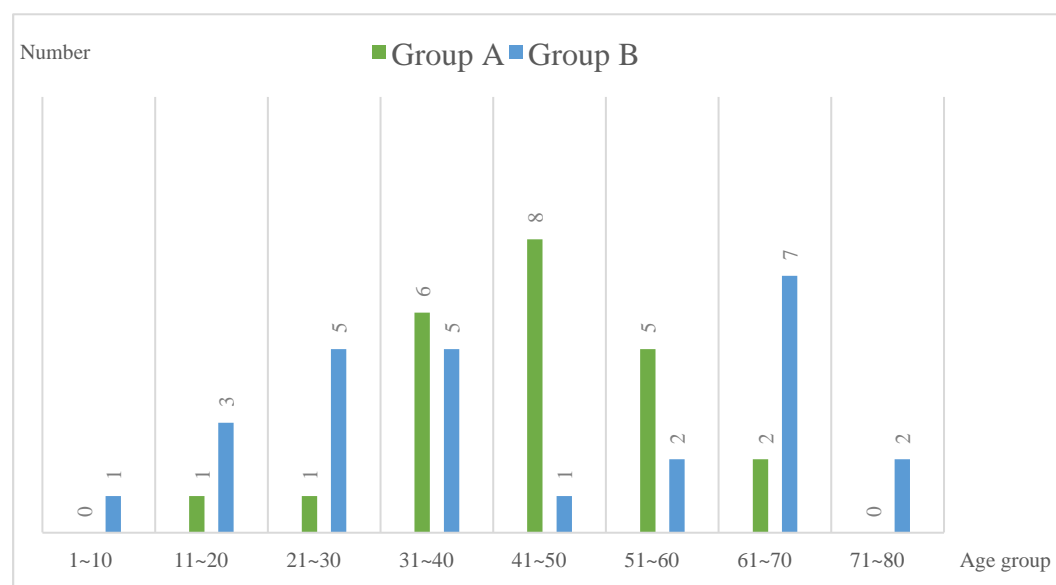

Figure S1. The age distribution of Group A and Group B.

Supplement: Supplementary file 1 [file Image_1.pdf]
