# Supplementary material for: Clinical features and prognosis of patients with anti-GBM disease combined with mesangial IgA deposition
Source: Front Immunol. 2024 Jul 22;15:1373581. doi: 10.3389/fimmu.2024.1373581 (PMC11298365; doi:10.3389/fimmu.2024.1373581)
Supplement: Supplementary file 2 [file Image_2.pdf]

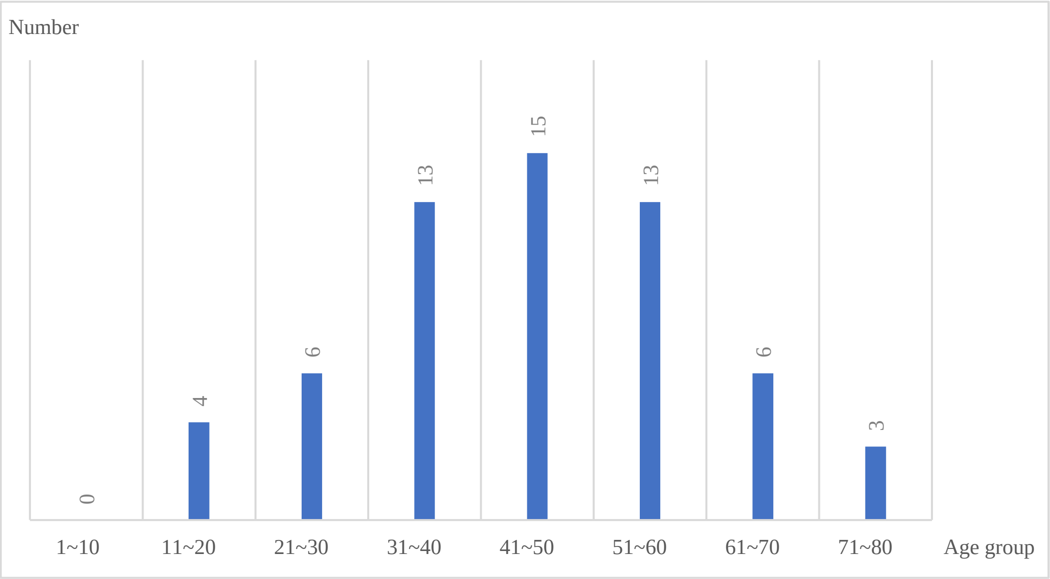

Figure S2.The age distribution of all reported patients with anti-GBM disease combined with mesangial IgA deposition.
